# Cholesterol granuloma in the choroid plexus of a cat

**DOI:** 10.1186/s12917-022-03358-6

**Published:** 2022-06-27

**Authors:** Rouven Wannemacher, Anna Knebel, Holger A. Volk, Florian Hansmann

**Affiliations:** 1 Department of Pathology, University of Veterinary Medicine Hannover, Foundation, Bünteweg 17, 30559 Hannover, Germany; 2 Department of Small Animal Medicine and Surgery, University of Veterinary Medicine Hannover, Foundation, Bünteweg 9, 30559 Hannover, Germany; 3 Institute for Veterinary Pathology, Veterinary Faculty, Leipzig University, An den Tierkliniken 33, 04103 Leipzig, Germany

**Keywords:** Cholesterol granuloma, Choroid plexus, Feline, Magnet resonance imaging, Hydrocephalus

## Abstract

**Background:**

This case report describes the clinical presentation, magnetic resonance imaging (MRI) as well as the histopathological findings in an elderly cat with an intracranial cholesterol granuloma.

**Case presentation:**

An 11.5-year-old, male neutered cat was presented at the emergency service with deteriorating behavioral changes including aggression, and progressive generalized ataxia. Magnetic resonance imaging (MRI) of the brain revealed a large, well demarcated, inhomogeneous and contrast enhancing mass in the lateral ventricles with marked mass effect. Due to a poor clinical prognosis, the cat was euthanized. Histological examination identified the mass as a bilateral cholesterol granuloma in the choroid plexus of the lateral ventricles.

**Conclusions:**

Although plexus cholesterol granulomas are rarely seen in cats, they should be considered as a differential diagnosis in elderly patients with neurological signs such as behavioral changes.

## Background

Diagnosis of an intracranial mass in adult cats is frequently considered to be a neoplasia [1]. Especially MRI findings of a single intracranial lesion with mass effect are strongly correlated with neoplasia [1]. In general, the most common intracranial neoplasms in cats, dogs and humans are meningiomas [2]. Important differential diagnoses to be considered are intraventricular papillomas, adenomas and carcinomas of the choroid plexus [2, 3], lymphoma, cholesteaterol granuloma and non-neoplastic inflammatory lesions [4–6].

Despite the misnomer, cholesteatomas cannot be considered a neoplastic lesion. The term “Cholesterol granuloma” represents a significantly better description of the lesion. The pathogenesis of cholesterol granulomas development is only partly understood. According to previous publications, cholesterol granulomas arise secondary to repeated hemorrhage or chronic inflammation, leading to a deposition of cholesterol crystals with subsequent granulomatous inflammation foreign body reaction [7, 8]. In horses, intracranial cholesterol granulomas frequently arise in the choroid plexus and are more frequently described than in cats or other species (Table 1) [7–11]. In the majority of cases, they affect the third or fourth ventricle (Table 1) and usually remain without clinical significance [8, 12].

**Table 1:** Reported occurrence of intracranial cholesterol granulomas in different species with presumably predisposing lesions. Intracranial cholesterol graulomas are most frequently described in horses. In cats, intra-axial cholesterol granulomas are commonly associated with meningioma

Species	Number of animals	Localization	Associated lesion	Reference
cat	1	Forebrain	Meningioma	[12]
cat	1	Extra-axially, falx cerebri	-	[4]
cat	1	Choroid plexus	Meningioma	[13]
cat	1	Extra-axially, falx cerebri	-	[14]
cat	1	Third ventricle	Meningioma	[15]
horse	3	Choroid plexus	-	[8]
horse	1	Lateral ventricles	-	[11]
horse	1	Lateral ventricles	-	[10]
horse	4	Lateral ventricles	-	[9]
dog	1	Intracranial	-	[16]
dog	1	Intracranial	-	[17]

Cholesterol granulomas in cats and dogs most frequently occur in the middle ear because of chronic inflammation [18–22]. Intracranial cholesterol granuloma represents a rare finding in cats [4, 12–15]. Described localizations of cholesterol granulomas in cats include extra-axially in the subarachnoid space [14] or in the *falx cerebri* [4, 12]. In one of these cases, a cholesterol granuloma was associated with a colocalized meningioma [12]. Another study described a cholesterol granuloma of the choroid plexus of the third ventricle, associated with a colocalized meningioma in a cat [13]. The present study describes a case of a cholesterol granuloma of the choroid plexus without an associated primary lesion in an adult, male-neutered cat with associated clinical findings.

## Case presentation

An 11.5-year-old, male neutered European Short Hair Cat, was presented at the emergency service of the Clinic for Small Animals (University of Veterinary Medicine Hannover, Germany) due to behavioral changes (especially aggression), generalized ataxia and sneezing. For approximately 3 months, the cat was sneezing and for seven days prior to presentation treated for otitis externa with amoxicillin/clavulanic acid by the referring veterinary surgeon. The clinical signs deteriorated further. The referring veterinary surgeon performed then a dental restoration with the suspicion of inflamed and painful teeth as the cause for the clinical signs. The cat became more aggressive, showed episodes of ‘running fits’, uncoordinated scratching and generalized ataxia. The cat also started to urinate and defecate outside the cat´s litter box, was falling into the feeding dish, developed difficulties while jumping and finally showed compulsive pacing. Otherwise, the cat was regularly vaccinated and dewormed, kept as an indoor-cat and received a commercial diet.

During the physical and neurological examination, the cat was highly aggressive and defensive but otherwise alert. Pulse (144 beats per min), respiration (36 breaths per min), body temperature (37.9 °C), and other physical findings were considered normal. Because of hiding and avoiding walking, it was not possible to investigate whether ataxia or paresis were present. Otherwise, the neurological examination was unremarkable. Based on the history and the results of the clinical examination a forebrain lesion was suspected. Hematology, biochemistry, electrolytes, and thyroxine levels taken at the referring veterinary surgeon were unremarkable. Based on previous results, MRI was recommended for further diagnostic examination. MRI of the brain was performed with a 3 Tesla MRI unit (Phillips Achieva Medical Systems, Eindhoven, the Netherlands). MRI sequences included: T2-weighted images in transverse (tra) and sagittal (sag) plane, tra FLAIR (Fluid-attenuated inversion recovery), tra GRASE (Gradient and Spin echo), dorsal (dor) SPAIR (Spectral attenuated Inversion Recovery) as well as a tra, sag and dor T1-weighted pre- and post-contrast (Gadoteric acid/Gadoterate meglumine). Previously, the cat was anesthetized and placed in sternal recumbency for image acquisition. General anesthesia was induced with intramuscular dexmedetomidine (5 μg/kg), butorphanol (0.3 mg/kg) and midazolam (0.3 mg/kg) and intravenous alfaxalone dose to effect. After intubation, anesthesia was maintained with isoflurane in air and oxygen.

**Fig. 1:**
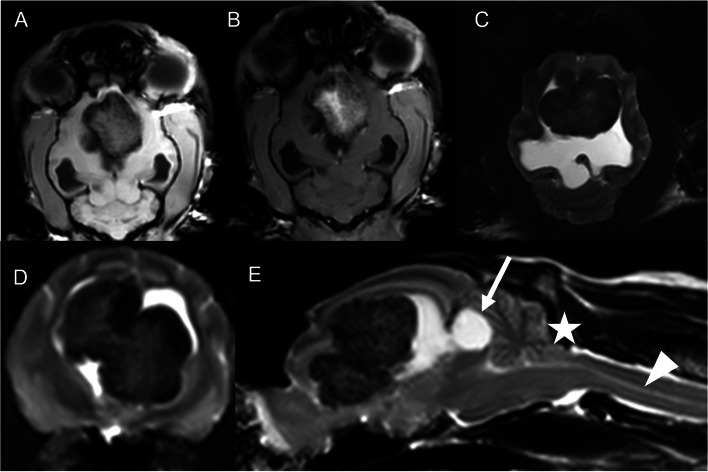
Dorsal (**A**, **B**, **C**), transverse (**D**) and sagittal (**E**) magnet resonance imagines of the brain with an intraventricular mass in a cat. The images represent as follows: **A** dorsal T1-weighted pre-contrast image, **B** dorsal T1-weighted post-contrast image, **C** dorsal T2 SPAIR image, **D** transverse GRASE image and **E** sagittal T2-weighted image. A focal, intra-ventricular, well demarcated, moderate to highly heterogeneous mass with inhomogeneous moderate to marked contrast enhancement is seen. This mass is heterogeneous, mostly hypointense on T1-weighted, T2-weighted, T2 SPAIR and GRASE images with hyperintensities and contrast enhancement mainly within the center of the mass (**A**-**E**). The space occupying intraventricular mass, leads to compression of the remaining brain parenchyma, internal hydrocephalus, marked supracollicular cerebrospinal fluid accumulation (**E**, white arrow), cerebellar herniation (**E**, white asterisk) and hydromyelia (**E**, white arrowhead)

The MRI revealed a large, focal, extra-axial, lobulated, well demarcated, moderate- to highly heterogeneous mass within the lateral ventricles. This heterogeneous mass lesion was mostly hypointense to surrounding brain parenchyma on T1 as well as T2-weighted images, SPAIR and GRASE. Hyperintensities were mainly found within central parts of the mass and the mass was inhomogeneous, moderate to marked contrast enhancing (Fig. 1). The space occupying, intraventricular mass consisted of two bilateral slightly asymmetric halves within the ventricles and therefore leading to a marked dilation of both lateral ventricles and marked mass effect, including compression of the remaining brain parenchyma, internal hydrocephalus, marked supracollicular cerebrospinal fluid accumulation and moderate to severe cerebellar herniation (Fig. 1). Furthermore, the cavity of the olfactory bulb and the central canal of the spinal cord was dilated (hydromyelia), an alteration of activity of the choroid plexus and therefore cerebrospinal fluid production and physiology was therefore suspected. Based on the previous description of the MRI findings, a cholesterol granuloma with bleedings was considered. The cat was euthanized for animal welfare reasons because of the guarded prognosis and therefore no investigation of the cerebrospinal fluid was performed.

**Fig. 2:**
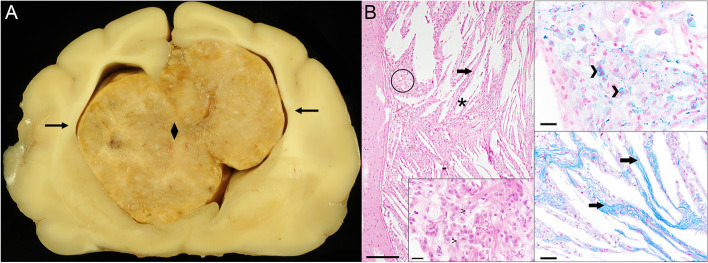
**A** Transversal brain section showing a 2.0 × 2.0 × 2.5 cm in size measuring mass (♦), almost completely filling the dilated lateral ventricles with disruption of the interventricular septum and compression of the adjacent parenchyma (➔). **B** Histopathology of the ventricular mass revealed a granulomatous inflammation with high numbers of foamy, hemosiderin-laden macrophages (O, > in insert) in the choroid plexus with numerous, optically empty “cholesterol clefts” (*), separated by fibrous septae (➔), bars represent 200 µm in the overview, 20 µm in the Prussian blue staining (top right) and the HE (bottom middle) as well as 40 µm in the Azan (bottom right)

At necropsy, a 2.0 × 2.0 × 2.5 cm large, bilateral, yellowish-grey, intraventricular mass with sharp borders causing an asymmetry of the brain parenchyma was found. The mass almost completely occluded both lateral ventricles, severely compressed the surrounding cerebral parenchyma (Fig. 2) and was associated with a mild internal hydrocephalus. Representative samples from cerebrum, cerebellum, brain stem, pancreas, kidneys, tonsils, lung, spleen, liver, bone marrow, diaphragm, tongue, nose, trachea, urine bladder, stomach, small intestine, large intestine, cerebellum, brain stem, cervical-, thoracic-, and lumbar spinal cord as well as thyroid glands were fixed overnight in 10% buffered formalin, paraffin-embedded, sectioned and stained with hematoxylin and eosin (HE).

Histologically, an expansive growing mass within the lateral ventricles extending from the choroid plexus was detected. The mass consisted of numerous, partly foamy macrophages with intracytoplasmic brown pigment as well as a deposition of cholesterol, characterized by optically empty, crystal-shaped clefts (“cholesterol clefts”), separated by septae of fibrous connective tissue (Fig. 2). By Prussian blue staining the intrahistiocytic pigment was identified to be hemosiderin, a product of hemoglobin degradation (Fig. 2). The morphological findings including granulomatous inflammation, cholesterol deposition and large quantities of hemosiderin-laden macrophages were consistent with a bilateral cholesterol granuloma of the choroid plexus of the lateral ventricles. The adjacent cerebral parenchyma was compressed and showed a mild to moderate gliosis. Within cerebellum and spinal cord no morphological alterations were detected. Additional histological findings included an oligofocal, moderate, lympho-plasmacytic pancreatitis and a multifocal, mild, non-purulent, chronic, interstitial nephritis.

## Discussion and conclusion

In general, intracranial masses rarely occur in cats [5, 23, 24]. The vast majority of clinically diagnosed intracranial masses in small animals are neoplasms with meningiomas and lymphomas representing the most common tumors described in cats [23, 25–28]. Differential diagnosis for intraventricular masses, as described in this case, include choroid plexus tumors (papilloma, adenoma or carcinoma), ependymoma or extra-axial lymphomas [23, 25–28]. In contrast, choroid plexus tumors are extremely rare in cats, accounting for only 0.4% of described intracranial neoplasms [23]. Depending on their cellular atypia, invasive growth and cerebral and spinal metastasis, they can be further categorized as choroid plexus papilloma or carcinoma [2, 3, 29]. Meningioma is by far the most frequent primary brain tumor in cats, accounting for approximately 58% of all intracranial neoplasms [23]. Meningiomas arise from meningothelial cells [30] and are especially common in areas around the venous sinuses [31]. In cats, they can occur as multiple meningiomas [23, 31] and are sometimes associated with cholesterol granuloma [12, 13, 15]. In fact, many currently described cases of intracranial cholesterol granuloma in cats were associated with a meningioma [12, 13, 15] suggesting that the meningioma may represent a possible trigger for the development of a cholesterol granuloma.

This case report describes the rare case of an intraventricular cholesterol granuloma without a detectable primary neoplastic or inflammatory insult in a cat. Primary non-neoplastic intracranial masses are extremely rare in cats [1]. When occurring, they are most likely infectious in origin [5, 24]. There have been few described cases of intracranial granuloma following an infection with *Cryptococcus neoformans* [5] or *Toxoplasma gondii* [24]. Also, an infection with the *Feline Infectious Peritonitis Virus* (FIPV) can lead to pyogranulomatous meningoencephalomyelitis and chorioiditis [32]. Cholesterol granuloma without an associated meningioma has only been reported in two other cases where it occurred extra-axially in the area of the *falx cerebri* [4, 14]. Other than intracranially, cholesterol granulomas in cats have been described in the middle ear, in association with chronic otitis media [33] and in the uterus, following chronic inflammation and repeated hemorrhage [34], indicating cholesterol granuloma as a most likely secondary lesion, occurring following a primary inflammatory, neoplastic or traumatic insult [35]. In the present case a primary lesion was not detected and therefore the occurrence of the cholesterol granuloma is suggested to be the primary lesion. The cholesterol granuloma in the choroid plexus, most likely by increasing the intracranial pressure and compression of the cerebral cortex, induced the observed clinical findings including altered consciousness ataxia and behavioral changes. The observed clinical findings were consistent with described changes observed in other animal species with expansively growing intracranial masses [23]. Future studies should investigate whether cholesterol granulomas in the ventricular system are spontaneously arising inflammatory lesions or part of a systemic, presently unidentified, feline pathogen.

## Data Availability

All data generated or analyzed during this study are included in this article.

## Abbreviations

MRI: Magnet Resonance Imaging
Tra: Traverse
Sag: Sagittal
FLAIR: Fluid-attenuated inversion recovery
GRASE: Gradient and Spin echo
Dor: Dorsal
SPAIR: Spectral attenuated Inversion Recovery
HE: Hematoxylin and eosin
FIPV: Feline Infectious Peritonitis Virus
